# Much More Than a Cytoskeletal Protein: Physiological and Pathological Functions of the Non-microtubule Binding Region of Tau

**DOI:** 10.3389/fneur.2020.590059

**Published:** 2020-10-19

**Authors:** Roland Brandt, Nataliya I. Trushina, Lidia Bakota

**Affiliations:** ^1^Department of Neurobiology, University of Osnabrück, Osnabrück, Germany; ^2^Center for Cellular Nanoanalytics, University of Osnabrück, Osnabrück, Germany; ^3^Institute of Cognitive Science, University of Osnabrück, Osnabrück, Germany

**Keywords:** Alzheimer's disease, membranes, microtubule-associated protein, tau, tauopathy

## Abstract

Tau protein (MAPT) is classified as a microtubule-associated protein (MAP) and is believed to regulate the axonal microtubule arrangement. It belongs to the tau/MAP2/MAP4 family of MAPs that have a similar microtubule binding region at their carboxy-terminal half. In tauopathies, such as Alzheimer's disease, tau is distributed more in the somatodendritic compartment, where it aggregates into filamentous structures, the formation of which correlates with cognitive impairments in patients. While microtubules are the dominant interaction partners of tau under physiological conditions, tau has many additional interaction partners that can contribute to its physiological and pathological role. In particular, the amino-terminal non-microtubule binding domain (N-terminal projection region, NTR) of tau interacts with many partners that are involved in membrane organization. The NTR contains intrinsically disordered regions (IDRs) that show a strong evolutionary increase in the disorder and may have been the basis for the development of new, tau-specific interactions. In this review we discuss the functional organization of the tau protein and the special features of the tau non-microtubule binding region also in the connection with the results of Tau KO models. We consider possible physiological and pathological functions of tau's non-microtubule interactions, which could indicate that interactions mediated by tau's NTR and regulated by far-reaching functional interactions of the PRR and the extreme C-terminus of tau contribute to the pathological processes.

## Introduction

Tau is a neuronal microtubule-associated protein (MAP) that is thought to be involved in the regulation of axonal microtubule assembly. In the human genome, tau is encoded by a single gene on chromosome 17q21 (1) and is expressed in several alternatively spliced isoforms. In the human central nervous system (CNS), six different isoforms are present, which differ by the presence or absence of three alternatively spliced exons, exon 2, 3, and 10 (2). The longest isoform in the CNS is encoded by 11 exons and contains 441 amino acids. In the peripheral nervous system (PNS) and the retina (retinal ganglion cells), also longer tau isoforms are present (3).

Tau belongs to a family of structural MAPs that share a conserved carboxy-terminal domain containing the microtubule-binding region (MBR). Besides tau, the family includes the neuronal MAP2 and the non-neuronal MAP4. Bioinformatics analysis indicates that the genes of the two neuronal MAPs, tau and MAP2, are the result of a gene duplication event that occurred at the dawn of the vertebrates (4). Since tau is enriched in the axon while MAP2 is mainly localized to the somatodendritic part of neurons, the N-terminal regions of the two proteins may provide the specific interactions, which are involved in the differential subcellular localization of the two MAPs. During neuronal development, tau localizes early to the axonal compartment, whereas MAP2 becomes restricted to the somatodendritic compartment at a later stage, at least in cultured neurons (5–7).

During Alzheimer's disease (AD) and other tauopathies, tau aggregates in the somatodendritic compartment into neurofibrillary tangles (NFTs) composed of tau proteins with increased stoichiometry of phosphorylation (“hyperphosphorylation”) (8). Dysregulation of tau splicing, increased expression of longer tau isoforms containing exon 10, and disease-like tau modifications have been shown to be associated with the development of tauopathies indicating a major role of changes in tau expression and post-translational modifications for disease development (9–11). It is noteworthy that the lack of tau protein only causes subtle changes in the corresponding mouse models and in particular does not have a major influence on the stability of the axonal microtubules. Even acute inactivation of tau in cultured nerve cells does not affect the stability of neuronal microtubules (12). Thus, the data strongly indicates that tau acts not as a stabilizer of microtubules in the axon contrary to the view, which is still quite common in the literature, but instead contributes to regulating microtubule dynamicity (13). On the other hand, the lack of tau reduces pathological changes in various mouse models of AD, stress, excitotoxicity and autism (14–17). This could indicate a “gain of function” mechanism of tau at pathological conditions that is not associated with its role as a microtubule-modulating protein. Bioinformatics analysis showed a minimal interactome of 73 direct binding partners (18), and 175 potential new and known tau interacting proteins were recently identified by MALDI-TOF mass spectrometry (19). Thus, tau appears to be a multifunctional protein with many interaction partners, and pathological changes in its interactome could contribute significantly to disease development in AD and other tauopathies.

**Figure 1:**
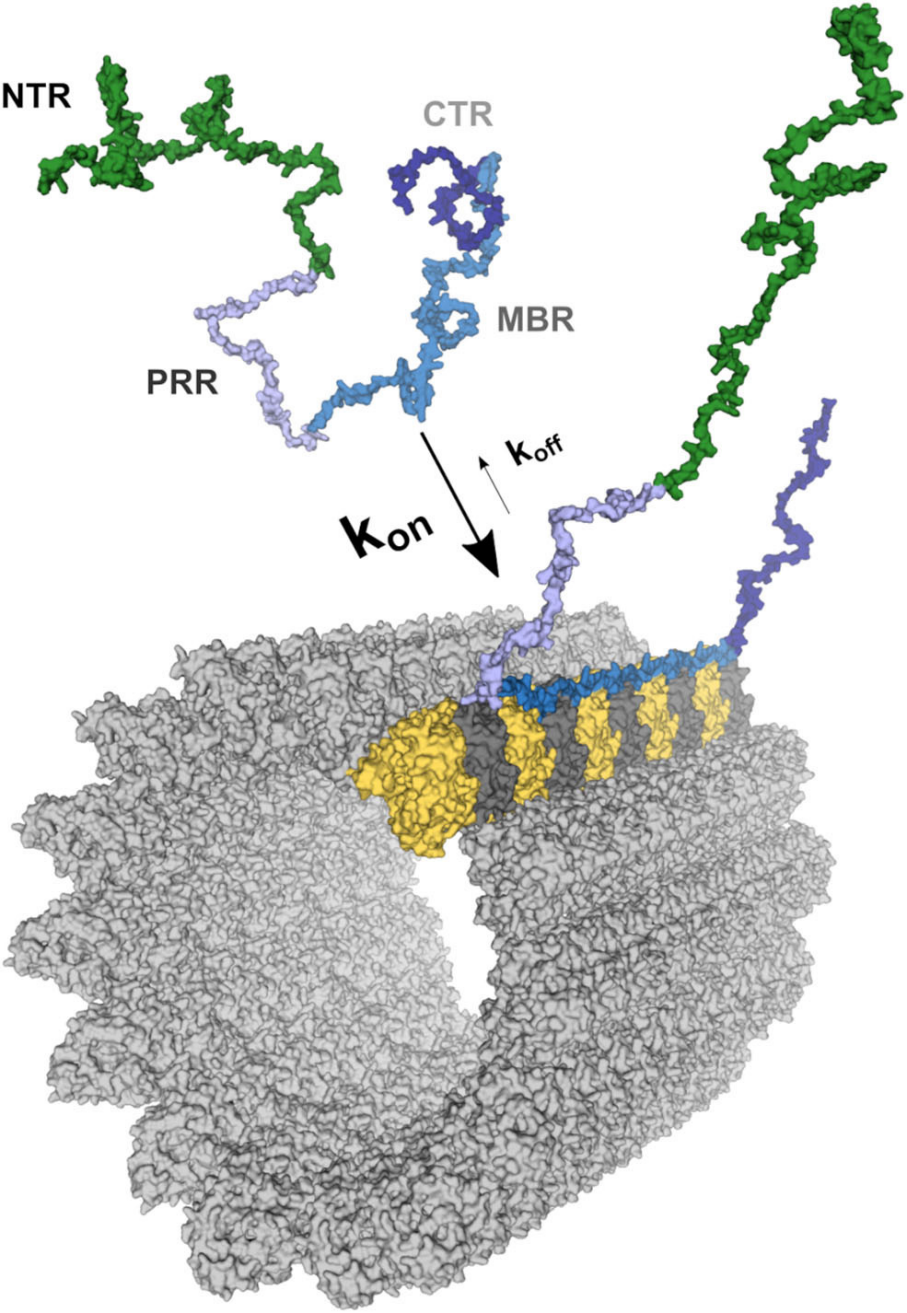
Schematic representation of the tau-microtubule interaction. A free molecule of tau is represented as one of the potential conformations of tau (441 aa long CNS isoform) generated as described previously (20). The different tau regions were mapped onto the model and color-coded as follows: NTR (aa 1–171)—green; PRR (aa 172–243)—light blue; MBR (aa 244–368)—blue; CTR (aa 369–441)—dark blue. The structure of the MBR binding to microtubules is based on PDB:6CVJ and PDB:6CVN structures showing interactions of first two microtubule-binding repeats of tau, R1 and R2, respectively (21). Further repeats, R3 and R4, were based on PDB:6CVN. The rest of tau molecule was artistically rendered based on the free molecule of tau. Binding to a single protofilament of a microtubule segment is depicted. α-tubulin is shown in yellow and β-tubulin in dark gray. All 3D structures are represented as surfaces and were visualized and rendered using PyMOL (www.pymol.org).

**Figure 2:**
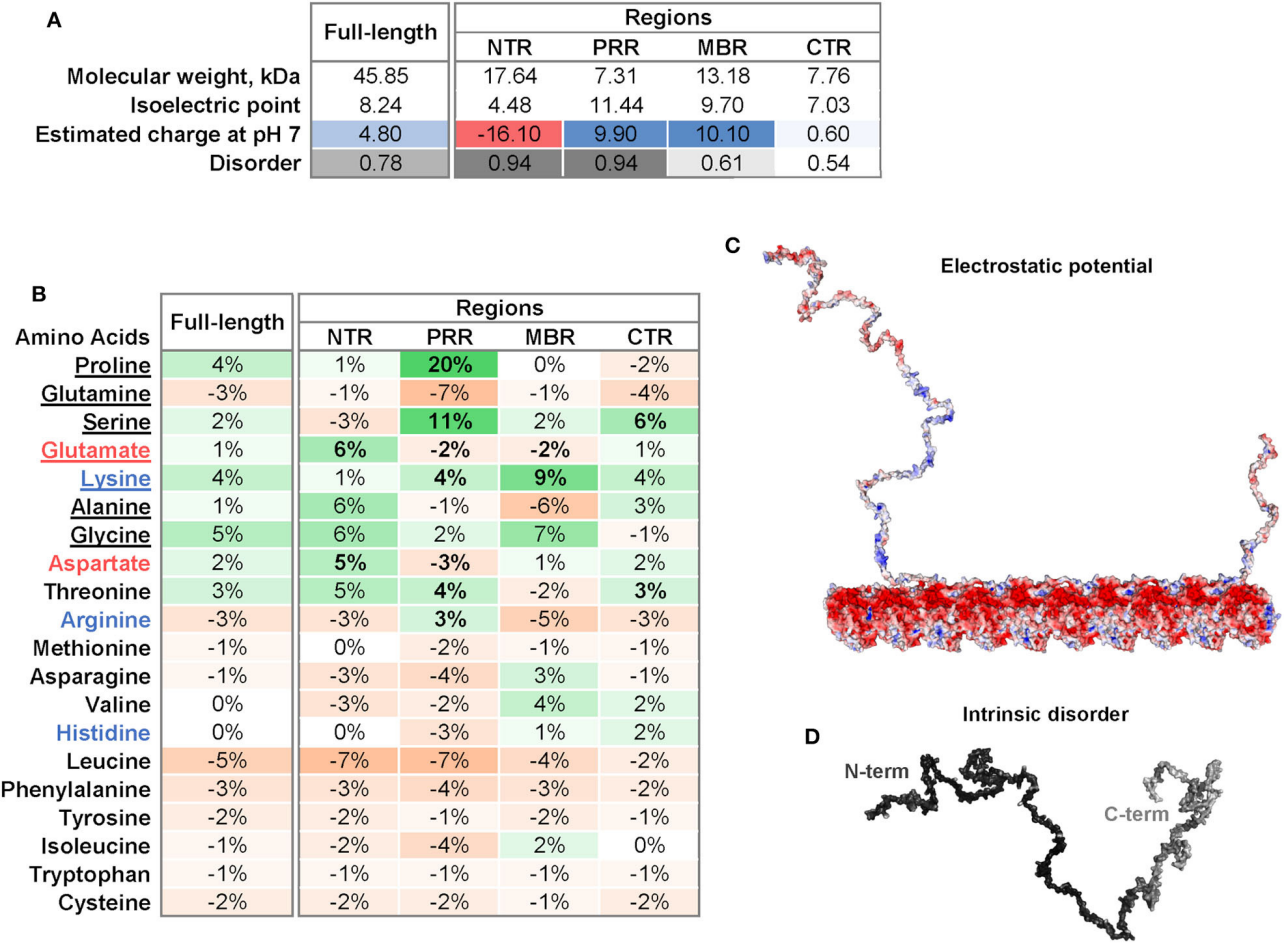
Physicochemical properties of tau and individual tau regions. **(A)** Isoelectric point, charge and disorder of full-length tau and the NTR, PRR, MBR, and CTR. The estimated charge at pH 7 is color-coded from acidic (red) to basic (blue). The extent of disorder is indicated as gray value. **(B)** Differences in frequencies of amino acids in tau and its four regions compared to the frequencies of amino acids encoded by the human genome according to (22). Overrepresented values are indicated in green, underrepresented values in orange color. Amino acids are sorted from disorder-promoting to order-promoting; disorder-promoting amino acids are underlined (23). **(C)** Electrostatic potential of tau and a microtubule protofilament. Electrostatic surface of tau was calculated with APBS Electrostatics Plugin (24). The electrostatic potential is color coded from red (negative) to blue (positive) at physiological pH. **(D)** Extent of intrinsic disorder of tau. Disorder was predicted using IUPred2A long (25). The extent of disorder was mapped onto the tau surface and indicated by white to black values as low or high disorder prediction, respectively.

Four regions of tau protein can be distinguished on the basis of sequence properties, the N-terminal projection region (NTR), the proline-rich region (PRR), the microtubule-binding region (MBR) and the carboxy-terminal region (CTR) (Figure 1). In particular, the NTR protrudes from the microtubule surface when tau is bound to microtubules and could mediate interactions with other cellular components. The NTR is acidic and highly negatively charged at physiological pH, while the PRR and the MBR are both very basic (Figures 2A,C). A striking feature that distinguishes the regions is also the level of disorder. Intrinsically disordered regions (IDRs) are characterized by the presence of low sequence complexity and amino acid compositional bias (26, 27). Functionally, IDRs could be relevant because they offer a large interaction area, are known to interact with many binding partners, and are involved in cellular signaling and regulation processes (18). The extent of disorder is highest for the NTR and PRR where disorder-promoting residues except glutamine are overrepresented (Figure 2B) (23). This indicates that in particular the non-microtubule binding part of tau has a high degree of binding promiscuity (Figures 2A,D). It is noteworthy that the disorder extent of the NTR and PRR shows a strong increase during vertebrate evolution, while the MBR and CTR showed a negative trend (20).

**Figure 3:**
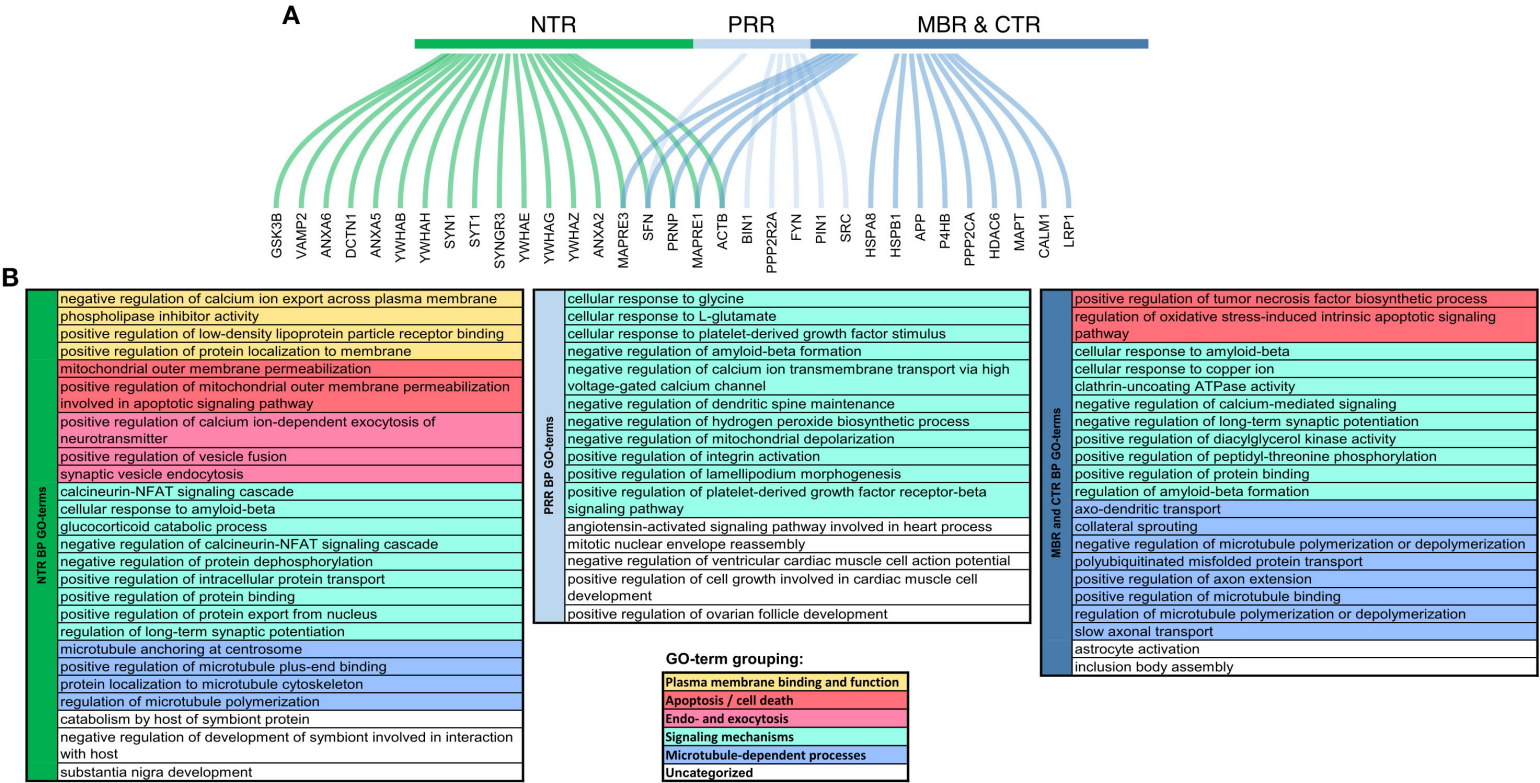
Functional specialization of tau regions with respect to their molecular interactions. **(A)** Genes coding for interaction partners of specific tau regions. Curved lines show to which region of tau the interaction partner was mapped colored by regions, respectively. NTR (1–171), green; PRR (172–243), light blue; MBR and CTR together (244–441), blue. **(B)** Summary representation of GO-terms for the interaction partners, which have been mapped to the different tau regions. GO-term enrichment was performed by ClueGO plugin in Cytoscape (29, 30). Significantly enriched GO-terms (pV < 0.05) associated with Biological Processes were identified using a right-sided hypergeometric test with Bonferroni correction. GO-term fusion was used to obtain the more representative terms. GO-terms were grouped and color-coded as presented in the legend below.

The projection domain of tau's neural relative, MAP2, also showed some regions of disorder, but displayed no apparent trend in their change during evolution (28). The organization and the different evolution of the tau sequence therefore suggest that tau is a multifunctional protein in which the N-terminus and the C-terminus have different interactions and contribute to different signaling events that are probably relevant for its physiological and pathological role. In fact, interactions between different tau regions with many different proteins have been found, which showed a certain degree of overlap. However, also several interactions, which are specific for certain regions, particularly with respect to the NTR and the MBR were described (Figure 3).

In this article, we want to examine the hypothesis that the different regions of tau have distinct interactions and are involved in various physiological functions and pathological alterations. To this end, we are evaluating data on the functional interactions mediated by the different regions of the tau protein, and are also taking a closer look at studies of tau knockout animals that could provide clues to non-microtubule-related functions of tau. In addition, we discuss the role of possible pathological changes in functional interactions that are mediated by the different tau regions.

## Functional Interactions of Different Tau Regions

### Tau as a Microtubule Binding Protein: The MBR and CTR

Tau is an abundant protein in neurons of vertebrates where it is enriched in the axonal compartment at physiological conditions. Microtubules are the dominant interaction partner of tau, with the majority of the tau population being bound to cellular microtubules (31, 32). However, the interaction of tau with axonal microtubules is remarkably dynamic (33). Tau rapidly binds and detaches from the microtubule surface in cultured neurons, a feature that has been termed “kiss and hop,” with a mean dwell time of single tau molecules in the millisecond range (Figure 1) (34).

Tau's microtubule-binding region consists of three or four repeat regions (RRs) dependent on the presence of the alternatively spliced exon 10. Binding to microtubules occurs via small groups of evolutionary conserved residues (35–37). The MBR is highly positively charged at physiological pH due to an overrepresentation of lysine residues (Figures 2A,B). On the other hand, microtubules are negatively charged on their surface, which is mostly due to the exposed acidic glutamate-rich C-terminal regions of tubulin (E-hooks) (Figure 2C) (38, 39). Thus, electrostatic interactions of the positively charged MBR with the E-hooks is likely to play an important role in the tau microtubule interaction. This is also consistent with a recent near-atomic model of microtubule-tau interactions in which tau follows the ridge on the microtubule surface defined by the H11 and H12 helices of α-tubulin (Figure 1) (21). Such an exposed binding site is also consistent with the highly dynamic interaction of tau with microtubules and the short dwell time (34).

In addition to the RRs, a highly conserved pseudorepeat region (PRR) following the RRs on the C-terminal side strongly contributes to microtubule binding *in vitro* and in cells (37, 40). The PRR is also present in the other members of the tau/MAP2/MAP4 family suggesting that it acts as a general modulator of the microtubule interaction in all members of this protein family (40).

Post-translational modifications of tau, in particular phosphorylation, affect tau's binding to microtubules. The phosphorylation of serine 262, which is located in the MBR and forms hydrogen bonds with α-tubulin Glu434, greatly reduces the binding of tau to microtubules (21, 41). However, most phosphorylation sites that affect tau's microtubule interaction are located in two regions that flank the MBR at both sites (20). Most of these sites are present in the PRR and the CTR, which show a clear overrepresentation of serine residues followed by threonine (Figure 2). Another post-translational modification of tau with potential functional relevance is acetylation. Lysine residues are typical spots for acetylation and are highly overrepresented in the MBR. Acetylation leads to charge neutralization and has been shown to impair the interaction of tau with microtubules (42). It has been shown that tau itself has acetyltransferase activity and can be acetylated through its own autocatalytic activity (43).

**Figure 4:**
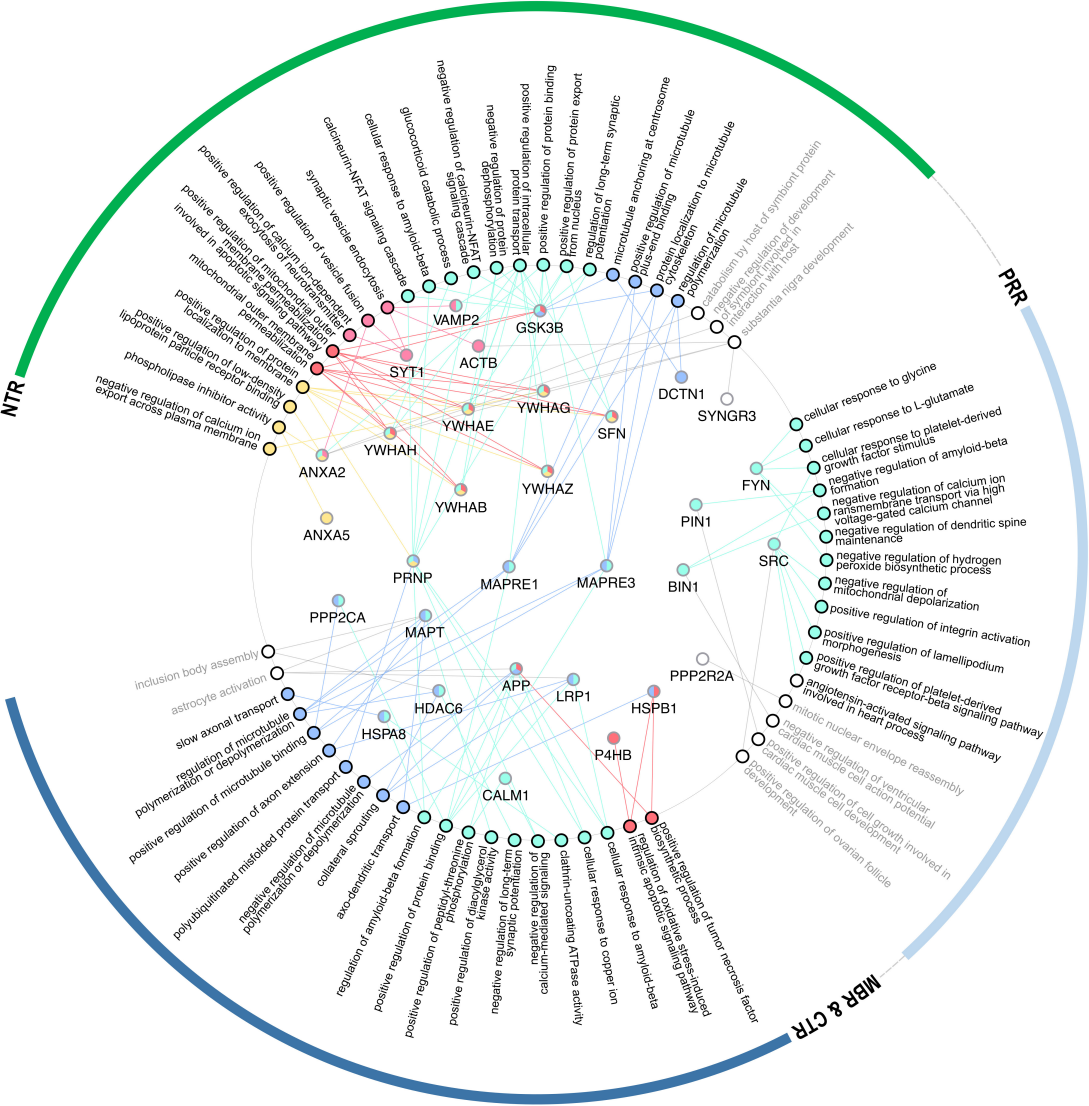
GO-term representation of the individual genes coding for interaction partners that have been mapped to bind to specific regions of tau. GO-terms were color-coded according to the groups as shown in Figure 3B.

Several binding partners have been identified, whose interactions have been mapped specifically to tau's MBR. In addition to microtubules these include heat shock proteins (44–46), tau itself (47), actin (48–50), and end-binding protein [EB2; (51)] [Figure 4; for a complete list with references see Table 1.1. in (20)]. A new addition to the proteins that interacts with tau's MBR is low-density lipoprotein receptor-related protein 1 (LRP1) which may be involved in the endocytosis of tau and its subsequent spread (52). The interaction of tau with LRP1 is mediated by lysine residues in the microtubule-binding repeat region of tau, which mainly contribute to the positive charge of the MBR (Figure 2B). Gene ontology (GO)-term grouping shows that the majority of the interacting proteins are associated with microtubule-dependent processes and signaling mechanisms. Few interactions also show associations with cell death mechanisms (Figure 3).

### Membrane-Related Functions of the Amino-Terminal Binding Domain of Tau

The most obvious peculiarity of the primary sequence of the N-terminal region of tau is a high content of aspartate and glutamate which makes this region highly acidic (Figures 2A,B). This is in marked contrast to the PRR and the MBR, which are both positively charged at physiological pH. Tau's NTR shows also the lowest degree of evolutionary conservation compared to the other tau regions (4) except two conserved sequence motifs in the extreme N-terminus, which may be involved in the interaction with annexins [amino acids 6–13 and 27–34; (53)].

GO-term groupings of the proteins, that have been mapped to specifically interact with the NTR reveals an enrichment of proteins implicated in plasma membrane binding and function, and endo-/exocytosis. The interaction of the NTR with a variety of membrane-associated proteins is a unique feature compared to the other regions of tau. Respective proteins include different annexins [A2, A5, and A6; (53, 54)], a multigene family of Ca^2+^-regulated membrane binding proteins that are thought to organize the interface between the cytoplasm and the cytoplasmic face of cellular membranes (55). Other membrane associated proteins are synaptic vesicle-associated proteins, such as Synapsin-1 and Synaptotagmin-1 (54), which modulate exocytosis and transmitter release (56). Recently, it has also been shown that tau's NTR interacts with synaptogyrin-3, an integral membrane protein of synaptic vesicles (57). For several interactions of the NTR with membrane components it has been observed that binding is phosphorylation-dependent indicating a potential regulation by signaling mechanisms. Generally, membrane-associated tau appears to be less phosphorylated than the cytosolic fraction (58–60). *In vitro*, also direct interaction of tau with lipids has been reported but it appears that these interactions are mainly mediated by the MBR and not the NTR since it involves short amphipathic helices located in tau's microtubule-binding repeats (61–65).

The GO-term grouping shows that some of the interacting proteins are also associated with signaling mechanisms. This includes, for example, glycogen synthase kinase 3 beta (GSK3β) (66), a proline-directed serine-threonine kinase that is involved in regulating neuronal survival, plasticity and metabolism (67). Tau itself is also a substrate for phosphorylation by GSK3β, which appears to be the main tau kinase *in vivo* (68). Other proteins in this group are several members of the 14-3-3 protein family (54, 69), which are highly expressed in the brain. 14-3-3 proteins are known to modulate the phosphorylation and interactions of many proteins thereby having the potential to modulate a variety of different cellular processes (70). However, it has also been found that some members of the 14-3-3 protein family interact with the PRR and MBR (71), indicating that this interaction may not contribute to the region-specific functions of tau.

### The Proline Rich Region as a Module Regulating Signaling

The proline rich region bridges the NTR with the MBR. As the name indicates, the PRR is characterized by a high relative content of proline (20% higher than the average distribution of amino acids in human proteins; Figure 2B), which is known for its exceptional conformational rigidity if incorporated in proteins. The PRR is positively charged at physiological pH and has the highest isoelectric point of all tau regions (Figure 2A). This is due to a particular high relative content of guanidinium groups (pKa of 12.5) due to the overrepresentation of arginine (Figure 2B). The PRR also has the highest relative content of serine making it tau's prime region for phosphorylation, followed by an above-average content of threonine. In fact, the PRR contains 22 predicted phosphorylation sites of which 14 are serine residues (NetPhos 3.1 Server, Technical University of Denmark).

GO-term groupings of the proteins that specifically interact with the PRR show a remarkable enrichment of proteins that are involved in signaling mechanisms. These include kinases, such as the src-family non-receptor tyrosine kinase fyn (72), which have also been shown to phosphorylate tau (73) and the protein phosphatase PP2A/Bα (74). Another interacting protein is the peptidyl-prolyl cis/trans isomerase NIMA-interacting 1 (Pin1), which can regulate the tau conformation and function (75, 76).

Phosphorylation within the PRR can affect both the interactions of the NTR and the MBR, suggesting that the PRR can act as a signaling module for the function of both regions. It has been shown that individual phosphorylation events in the PRR, e.g., phosphorylation of Ser-214 by cAMP-dependent protein kinase (PKA) reduces the interaction between tau and microtubules and suppresses *de novo* microtubule polymerization (77, 78). Phosphorylation-mimicking glutamate clusters in the proline-rich region had a similar effect on microtubules and were also sufficient to abolish the tau association with the plasma membrane (79).

## Lessons From Tau Knockout Animals

Analysis of knockout animals can provide important information on the function of a particular protein in a systemic context. Thus, we screened the literature for publications, where tau knockout animals have been characterized. A summary of the studies and key results are shown in Table 1. Many of the studies report changes in memory formation and anxiety-related behavior, which could be caused by many deficits, including changes in microtubule-dependent processes, endo- or exocytosis, signaling mechanisms, or plasma-membrane related functions.

**Table 1:** Summary and key results of studies using tau knockout animals.

**Tau KO animal**	**Experimental approach**	**Change in Tau KO animals**	**References**
Scn1aRX/+ and Cntnap2^−/−^ mice × Tau KO mice (Dawson)		Diminished epilepsy, abnormally enlarged brains, and overactivation of the phosphatidylinositol 3-kinase (PI3K)/Akt (protein kinase B).	(17)
Tau KO mouse		Impaired contextual and cued fear memory.	(80)
Tau KO mouse	Fluid percussion injury	Lower anxiety and improved motor function after recovery.	(81)
Tau KO mouse		Decrease in functional extrasynaptic NMDA receptors in the hippocampus.	(82)
Transgenic mouse model of α-synucleinopathy (TgA53T) × Tau KO		Ameliorates cognitive dysfunction and concurrent synaptic deficits of TgA53T mice.	(83)
Tau KO mice		Increased ATP production and improved recognition memory and attentive capacity of juvenile mice.	(84)
Tau KO mice		Olfactory deficit correlated with accumulation of α-synuclein and autophagic impairment.	(85)
Acute Tau KO		Impaired motor coordination and spatial memory.	(86)
Tau KO (tauΔex1)		Reduced susceptibility to excitotoxic seizures.	(87)
Tau KO on B6129PF3/J genetic background		Age-dependent short-term memory deficits, hyperactivity and synaptic plasticity defects.	(88)
Tau KO		Hyperglycemic and glucose intolerance; reduced islet insulin content and elevated proinsulin levels; increased epididymal fat mass and leptin levels; reduced glucose production, and insulin resistance at later ages, leading to complete onset of diabetes.	(89)
Transgenic (J20) mice express human amyloid precursor protein (hAPP) with the Swedish (K670N, M671L) and Indiana (V717F) mutations under the control of the PDGF β-chain promoter × Tau KO mice (Dawson)		Hippocampal hyperactivity, but not mPFC hypoactivity, is attenuated by deletion of tau; tau depletion failed to reverse the memory impairment induced by over-production of APP in MWM; deletion of tau alleviated the hyperlocomotion displayed by APP transgenics.	(90)
Tau KO	Experimental stroke, using a middle cerebral artery occlusion with reperfusion model	Protection from excitotoxic brain damage and neurological deficits.	(16)
Tau KO	Stress-driven suppression of neurogenesis	After exposure to chronic stress no reduction in DG proliferating cells, neuroblasts and newborn neurons.	(91)
Tau KO (Dawson)	Cortical cultures Treatment with extracellular tau	Much less affected transport of BDNF, BACE1 or NPY.	(92)
Tau KO (Dawson)		Heterozygous tau knockout, but not homozygous knockout, induced a selective loss of VTA DA neurons at the early post-natal stage P0, which correlated with a similar reduction in Otx2 expression and increases in prenatal cell death and the unactivated compensation effect of MAP1A in Mapt^+/−^ mice.	(93)
Tau KO (Dawson)	Unilateral, transient middle cerebral artery occlusion (MCAO)	Mice were protected against hemispheric reperfusion injury following MCAO at 3-months of age but not at 12-months.	(94)
Tau KO (Tucker)		Impaired hypothalamic anorexigenic effect of insulin that is associated with energy metabolism alterations.	(95)
Tau KO mice (Dawson)		Increased locomotor activity in 5-months-old animals compared to human wild-type expressing animals.	(96)
Tau KO mice		Tau ablation blocks stress-driven anxious, anhedonic, and passive coping behaviors as well as cognitive impairments; chronic unpredictable stress decreased NA and 5HT levels in WT, but not Tau-KO, animals; stress-driven structural remodeling of hippocampal neurons depends on tau protein.	(15)
Tau KO mice (Dawson)	Primary cultures of hippocampal neurons	Tau is required for normal interactions of RNA binding proteins in brain tissue and tau promotes stress granules, while TIA1 promotes tau misfolding and insolubility.	(97)
Tau KO mice (Dawson)		Tau facilitates kainic acid (KA)-induced seizures in vivo;tau facilitates ROS production in response to excitotoxic insult in vivo.	(98)
Tau KO mice	Stereotactic injection of Aβ42 oligomers into the hippocampal dorsal CA1 area bilaterally	Protection against Aβ-induced cognitive impairment, hippocampal neuron loss, and iron accumulation.	(99)
Tau KO mice	Primary cultures of cortical neurons	Protection of mouse primary cortical neurons from loss of mitochondrial membrane potential (ΔΨm) caused by low concentrations of Aβ42; absence of tau resulted in significantly greater $\text{increases in Ca}{}_{\text{cyt}}^{2+}$ in response to Aβ treatment.	(100)
Tau KO mice		Basal synaptic transmission of mossy fibers measured by input output curves is decreased; bouton diameter increase of ~45%.	(101)
Tau KO mice on Bl6/129sv and Bl6 backgrounds		Complete tau reduction impairs the performance of mice in accelerated Rotarod test, impairs the performance of mice in Pole test, impairs the performance of mice in Openfield test, alters hindlimb clasping behavior at 12 months of age; motor deficits are related to nigral degeneration.	(102)
Tau KO mice		Treatment with streptozotocin did not lead to impaired hippocampal cognitive behavior in Tau KO mice nor in reduction of PDS-95, synaptophysin and p-CREB.	(103)
Tau KO mice		Impaired LTD but not LTP in Tau KO mice at 7–11 months of age.	(104)
Tau KO mice	Primary neurons	Tau deficiency prevents AβO-induced polyglutamylation, spastin recruitment, and TTLL6 transport into dendrites; tau deficiency does not protect against Aβ association to dendrites and transient spine loss, but protects against loss of MTs, neurofilament invasion, and loss of mitochondria.	(105)
Tau KO mice (Dawson)	Primary cell culture	Tau is required in cultured neurons for ectopic cell cycle re-entry (CCR) induced by Aβ.	(106)
Tau KO mice (Dawson)		Subtle motor deficits at 12–15 months of age connected to mild dopaminergic deficits in Tau KO mice.	(107)
Tau KO mice (Dawson)		Tau-knockout mice develop age-dependent brain atrophy, iron accumulation and substantia nigra neuronal loss, with concomitant cognitive deficits and parkinsonism.	(108)
Tau KO mice (Dawson) and mice expressing APP with the Swedish mutation Tau KO background		Overexpression of mutant APP in tau knockout mice, elicits the extensive formation of axonal spheroids and more severe cognitive deficits.	(109)
Tau KO mice	Primary cultures of cortical neurons	A significantly lower LDH release, with a peak delayed by 24 h, was detected in Tau KO neurons after heat shock.	(110)
Tet/GSK3β mice on Tau KO (Dawson) background		The toxic effect of GSK3 overexpression is milder and slower in the absence of tau.	(111)
Tau KO mice (Dawson)		A deficit in migration of newborn cells in the subgranular zone was observed in Tau KO mice.	(112)
Tau KO mice	Primary cultures of hippocampal neurons	Tau-depleted neurons showed no signs of degeneration in the presence of Aβ.	(113)
Tau KO mice (Dawson)	Primary cultures of hippocampal neurons	Inhibition of neuronal maturation.	(114)
Tau KO mice (Harada)		Altered microtubule organization in small-caliber axons.	(115)

*The following color code was used to assign results to the summary of GO-terms introduced in Figure 3: yellow: plasma membrane binding and function; red: apoptosis/cell death; purple: endo- and exocytosis; green: signaling mechanisms; blue: microtubule-dependent processes*.

Only few studies report effects, which can be directly related to microtubule-dependent processes. In the first study with Tau KO mice, an altered microtubule organization in small-caliber axons was described (115). Other studies include a report that showed that neurons from Tau KO animals were less affected with respect to transport of Brain-derived neurotrophic factor (BDNF), Beta-secretase 1 (BACE1), or neuropeptide Y (NPY) after treatment with extracellular tau indicating that endogenous tau can be modified by exogenous tau in such a way that transport is impaired (92). In another study, it was observed that Tau KO protects against Aβ-induced loss of microtubules indicating that pathological conditions can cause tau to actively disassemble microtubules (105). It is however noteworthy that none of the studies report obvious destabilization or disassembly of microtubules in Tau KO animals indicating that stabilization of microtubules is not the primary function of tau in neurons.

Several studies report changes, which can be interpreted as being caused by disturbed plasma membrane binding and function. In one study, a decrease in functional extrasynaptic NMDA receptors was observed in Tau KO animals (82) suggesting a participation of tau in anchoring of specific transmembrane proteins of the post-synaptic plasma membrane. In another study, a significant increase in the diameter of synaptic boutons, small swellings that are found at the terminal ends of axons, was observed in Tau KO animals (101). This observation may also be related to a disturbance of plasma membrane organization. Interestingly, one study reports that overexpression of mutant amyloid precursor protein (APP) in Tau KO animals leads to extensive formation of axonal spheroids (109). Spheroids are axonal swellings with discontinuous or absence of myelin sheaths, which may indicate that tau stabilizes the organization of the axonal plasma membrane to support a functional interaction with oligodendrocytes.

Quite some studies report changes in processes, which could be related to disturbances in endo- or exocytosis. In one study hippocampal hyperactivity, which was induced by expression of APP with familial mutations, was found to be attenuated by depletion of tau (90). In another study it was reported that basal synaptic transmission in the hippocampus was decreased in Tau KO mice (101) and one study reports impaired LTD in Tau KO mice (104). In addition, several defects, such as reduced susceptibility to excitotoxic seizures (87), age-dependent hyperactivity and synaptic plasticity defects (88), or facilitation of kainic acid-induced seizures (98) that have been observed in Tau KO animals may be caused by changes in endo- or exocytosis. In fact, also some of the systemic effects, such as changes in memory- and anxiety-related behavior (80, 81, 86) could be interpreted as a result of impaired neurotransmitter exocytosis.

Interestingly, also effects on survival of specific cell populations have been reported. This includes a selective loss of dopaminergic neurons in the ventral tegmental area (VTA) and increased prenatal cell death (93), and degeneration of neurons of the Substantia nigra (102) in Tau KO animals. However, opposite effects have also been reported, in which the absence of tau promotes cell survival after toxic attacks. Examples are reports in which Tau KO protects against excitotoxic brain damage (16) or against Aβ-induced loss of hippocampal neurons (99).

Finally, also several reports support a role of tau in modulating signaling mechanisms. In particular it is striking that several papers report impaired glucose metabolism and response to insulin in Tau KO mice (89, 95). In addition, changes in ROS production (98) and Ca^2+^ homeostasis (100) in response to pathological conditions have been reported.

Thus, the results of the analysis of Tau-KO animals support a multifunctional role of Tau, whereby many of the changes observed, in particular with regard to a disturbance in the function and organization of the plasma membrane, to endocytosis and exocytosis, to signaling mechanisms and to the cell death mechanism, are very likely mediated by tau's N-terminal projection domain.

## Pathological Changes of Functional Interactions of the Different Tau Regions

Tau pathology is associated with a decreased binding of tau to axonal microtubules, a redistribution of tau from the axon to the somatodendritic compartment and aggregation of tau protein in NFTs. These events are joined by an increased phosphorylation of tau at selected sites and death of affected neurons. Since it is known that tau's interaction with microtubules is impaired by phosphorylation and familial tauopathy mutations, potential pathological changes of interactions mediated by tau's microtubule binding region were long in the focus of research. However, tau was also found to be present in a membrane-enriched proteome from post-mortem human brain tissue in AD (116) and has been implicated in the formation of toxic complexes with phospholipids (64), suggesting that pathologic changes in tau's membrane interaction mediated by its NTR may also have a central role in the disease process.

### Pathological Changes of the Interactions of the Microtubule Binding Region

Phosphorylation of tau at several sites is known to reduce the interaction with microtubules. Most of the regulatory sites are present in the PRR and the CTR, which flank tau's microtubule binding region on both sites. This is also reflected in PHFs that have been isolated from patients with AD, where all of the 10 major phosphorylation sites are located in the PRR and the CTR (20, 117). In addition, Ser262 (which is located in the MBR) has been identified as being phosphorylated in PHFs (118), but appears to be only weakly phosphorylated during the development of AD (119). Progressive phosphorylation during AD has been correlated best for the AT8 site (Ser202/Thr205) (120) but also Ser199 and Thr231, which belong to the major phosphorylated sites in PHFs, show an increase during progression of AD (119). All of these sites are located in the PRR and combined phosphomimicking mutations of five major PHF sites within the PRR (Ser198, Ser199, Ser202, Thr231, and Ser235) suppress de novo microtubule polymerization (79). Thus, a pathologic increase in tau phosphorylation at the PRR appears to impair tau's microtubule related activities. It should however be noted that a phosphomimicking tau construct of 10 major phosphorylation sites that have been identified in PHFs from AD was still able to interact with microtubules, albeit with a reduced dwell time (40). This indicates that phosphorylation-induced changes in tau's microtubule interaction may only partially account for the development of tau pathology, at least with respect to tau's redistribution and toxicity. This is in agreement with the view that tau is not a microtubule stabilizer in the neuron but a multifunctional MAP (121). It should also be noted that the PRR, where many of the sites with increased phosphorylation in AD are located, appears to act as a module that affects not only microtubule-related activities but may regulate also interactions and signaling events, which are mediated by the NTR. This is exemplified by the observation that pseudophosphorylation in the PRR abolished tau's interaction with plasma membrane components (59).

The MBR is also the region that mediates the tau-tau interaction, possibly influencing the formation of tau aggregates. The cores of PHFs and SFs from patients with AD are made of protofilaments comprising residues 306–378 of tau protein (122), which are located within the MBR and CTR. However, the role of phosphorylation in mediating tau aggregation remains unclear. While phosphorylation at different sites parallels tau aggregation in the brain (119), at least *in vitro*, site specific hyperphosphorylation inhibits, rather than promotes, tau fibrillization (123). However, pseudophosphorylated tau at Ser202 and Thr205 showed enhanced aggregation *in vitro* (124) suggesting that some sites have specific effects on filament formation. It is possible that other factors or modifications promote tau aggregation. The ratio of cis/trans isoforms may affect fibrillarization and *in vitro* data suggest that in particular the trans isomer of a tau peptide is prone to aggregate (125). Isomerization is regulated by the protein Pin1, which binds to the PRR of tau. Also, proteolytic degradation of tau may influence filament formation and several studies have provided evidence that tau cleavage by metalloproteinases, caspases, or a lysosomal cysteine proteinase induce tau aggregation (126–129).

Interaction with phospholipids or free fatty acids can also induce the aggregation of full-length tau (62, 130) or a tau fragment comprising the MBR (131). Interestingly, it has also been reported that the NTR can contribute to tau polymerization because a disease-associated R5L mutation in the NTR increases tau polymerization (132). The presence of an N-terminal tau fragment comprising the NTR and part of the PRR was also shown to inhibit tau aggregation, which confirms that the tau N-terminus plays a modulatory role in the aggregation process (133).

### Pathological Changes of the Membrane-Related Interactions of Tau

Evidence from cell-free experiments indicated that phospholipids induce changes in the tau conformation over the entire length of the molecule, which affect the phosphorylation of tau and its interaction with microtubules (134). Membranes facilitate tau aggregation *in vitro* (135), and it has been shown that tau binds to membranes via short amphipathic helices located in its microtubule binding repeats (63). In addition, pathological changes in membrane-related interactions of tau, mediated by the NTR that does not bind to microtubules, could play an important role in the redistribution of tau from the axon to the somatodendritic compartment and may also be involved in the induction of toxic effects. Tau's interaction with components of the plasma membrane is affected by mutations, which have been observed in familial forms of tauopathies, or by phosphorylation at disease-relevant sites. Interestingly, the Frontotemporal Dementia mutation R406W blocks tau's interaction with the membrane in an annexin A2-dependent manner indicating that changes in the C-terminus (mutation at position 406) affect an interaction, which is mediated by the extreme N-terminus (136). A similar observation was made with respect to phosphorylation, where pseudophosphorylation of disease-relevant sites at the C-terminal region blocked tau's membrane interaction (79). It appears surprising that changes in tau's carboxy-terminal region selectively affect a feature that is mediated by the opposite side of the molecule. However, it has been shown previously that tau can adopt a “paperclip” conformation, in which the amino- and carboxy-terminal domains approach each other (137) and MS analysis of the phosphorylation pattern have suggested that the R406W mutation exerts long-range conformational effects on the structure of tau (138). It should also be noted that although pseudophosphorylation in the carboxy terminus of tau abolished tau's interaction with the membrane cortex, tau's ability to promote microtubule assembly remained unchanged (79). This indicates that the interactions of the MBR and the NTR can be influenced differently by disease-associated phosphorylation events in different domains.

Increased phosphorylation as AD progressed was also observed at Tyr18, a site located at the extreme N-terminus of tau (119). Tyr18 is in the first exon of tau, which is also involved in the interaction with annexins, although this particular interaction does not appear to be affected by phosphorylation at this position (53). In the same region, familial tau mutations (R5H and R5L) were also observed in tauopathies (139, 140). In fact, phosphorylation at several tyrosine-residues including those in the N-terminus (Tyr18, Tyr29, and Tyr219) have very recently been shown to abolish tau aggregation and inhibit lipid-binding properties of tau *in vitro* (65).

Besides tau's interaction with plasma membrane components, it has also been shown that tau binds to presynaptic vesicles in AD patient brain. Human tau also interacts with synaptic vesicles in a Drosophila model and tau mutations, which are associated with familial forms of tauopathy, exhibit increased presynaptic localization and lead to reduced synaptic transmission (141). This suggests a presynaptic pathological role of tau, which is associated with a changed disease-associated interaction with synaptic vesicles mediated by the NTR of tau. Also, in this case, the familial mutations were located in the C-terminal part of tau (P301L, V337M, R406W) indicating that changes in the C-terminus can affect interactions, which are mediated by tau's NTR. The pathologic effect on synaptic vesicles may not be mediated via direct interaction with lipids since the same lab has identified the transmembrane vesicle protein synaptogyrin-3 as the binding partner of tau's N-terminus on synaptic vesicles (57).

It is also possible that tau oligomers, which have recently come into the focus as the primary toxic pathological tau species, affect membrane integrity and thereby exert toxic effects. In fact, it has been shown that tau oligomers affect the integrity of artificial phospholipid vesicles and decrease cell viability of a neuronal model (142), however it is unclear, whether membrane-binding properties of the NTR have a role in this process.

## Conclusions

Tau was originally identified as a microtubule-associated protein, but has been shown to have many additional interaction partners and can be considered a multifunctional protein. Indeed, tau knockout studies in mouse models show surprisingly small changes that may be directly related to impaired microtubule stability, impaired microtubule-dependent transport, or changes in microtubule dynamics. Therefore, non-microtubule-related functions mediated by the N-terminal region of tau could play an important role with regard to the functions and malfunctions of tau during tauopathies. There are several indications that are based on the identity of region-specific interaction partners or on the results of tau knockout studies that membrane-related functions of the NTR can disrupt the organization and function of the plasma membrane and other membrane organelles, such as synaptic vesicles. In addition, there are indications of disturbances in signal mechanisms and effects on the survival mechanisms of the cells, which may be mediated by the NTR. It is particularly noteworthy that post-translational modifications or disease-associated mutations in other regions of tau, such as the extreme C-terminus or the PRR, can affect the function of the NTR, suggesting that far-reaching functional interactions contribute to the pathological processes. Gain-in-function mechanisms mediated by the N-terminal region of tau could also explain the obvious paradox that knockout of tau, despite the long-standing (and probably incorrect) dogma of tau as a microtubule-stabilizing protein, has surprisingly few functional consequences in animal models and that the loss of tau can even increase resistance to stress and certain pathological conditions.

The sequence of the N-terminus of tau shows due to the presence of two alternatively spliced exons (exons 2 and 3) in CNS tau isoforms and other exons that are only present in PNS tau (e.g., 4A and 6) considerable variations (3). The PNS-specific exon 4A also shows considerable differences in length between humans, mice and rats. Therefore, it would also be important to gain a better understanding of the role of the N-terminal variants in terms of functional differences mediated by the regions of tau not related to binding to microtubules.

## Author Contributions

RB, NT, and LB interpreted the data and wrote the article. All authors contributed to the article and approved the submitted version.

## Conflict of Interest

The authors declare that the research was conducted in the absence of any commercial or financial relationships that could be construed as a potential conflict of interest.
